# Anti-Inflammatory Effects of Ginsenoside Rb3 in LPS-Induced Macrophages Through Direct Inhibition of TLR4 Signaling Pathway

**DOI:** 10.3389/fphar.2022.714554

**Published:** 2022-03-24

**Authors:** Honglin Xu, Min Liu, Guanghong Chen, Yuting Wu, Lingpeng Xie, Xin Han, Guoyong Zhang, Zhangbin Tan, Wenjun Ding, Huijie Fan, Hongmei Chen, Bin Liu, Yingchun Zhou

**Affiliations:** ^1^ Department of Traditional Chinese Medicine, Nanfang Hospital (ZengCheng Branch), School of Traditional Chinese Medicine, Southern Medical University, Guangzhou, China; ^2^ Guangdong Provincial Key Laboratory of Tropical Disease Research, Department of Pathogen Biology, School of Public Health, Southern Medical University, Guangzhou, China; ^3^ Department of Traditional Chinese Medicine, Binzhou Medical University Hospital, Binzhou, China; ^4^ Department of Traditional Chinese Medicine (Institute of Integration of Traditional and Western Medicine of Guangzhou Medical University, State Key Laboratory of Respiratory Disease), The Second Affiliated Hospital of Guangzhou Medical University, Guangzhou Medical University, Guangzhou, China; ^5^ TCM Health Construction Department of Yangjiang People’s Hospital, Yangjiang, China

**Keywords:** anti-inflammatory effects, ginsenoside Rb3, MAPK, NF-κB, TLR4

## Abstract

*Panax ginseng* has therapeutic effects on various inflammation-related diseases. Ginsenoside Rb3 (GRb3), a natural compound with anti-inflammatory and immunomodulatory properties, is one of the main active panaxadiol extracted from *Panax ginseng*. We explored whether GRb3 inhibited LPS-mediated inflammation through TLR4/NF-κB/MAPK signaling in macrophages. GRb3 attenuated NO and PGE_2_ production by attenuating iNOS and COX2 expression. GRb3 also suppressed pro-inflammatory cytokines levels, including IL-1β, IL-6, and TNF-α. Moreover, GRb3 administration significantly suppressed NF-κB (p65) nuclear translocation and the phosphorylation levels of p65, IκBα, JNK, p38, and ERK dose-dependently. Molecular docking demonstrated that GRb3 could dock onto the hydrophobic binding site of TLR4/MD2 complex, with a binding energy of −8.79 kcal/mol. Molecular dynamics (MD) displayed stable TLR4-MD2-GRb3 modeling. GRb3 dose-dependently inhibited LPS binding to cell membranes and blocked TLR4 expression. Surface plasmon resonance imaging (SPRi) revealed that GRb3 had an excellent binding affinity to TLR4/MD2 complex. Notably, resatorvid (TAK242), a selective TLR4 inhibitor, did not increase the repressive influence of GRb3 in RAW264.7 macrophages. Moreover, TLR4 overexpression partially reversed the repressive roles of GRb3 on the NF-κB/MAPK pathway and inflammatory mediators. Collectively, our study strongly indicated that GRb3 attenuated LPS-mediated inflammation through direct inhibition of TLR4 signaling. A novel insight into the underlying mechanism of anti-inflammatory effects of GRb3 in macrophages was confirmed.

## Introduction

Inflammation, as the self-defense mechanism of the body, is a physiological process against external pathogenic infection and cellular destruction, which may contribute to restoring the natural balance in the organism (Kotas and Medzhitov, 2015). Generally, the body regulates inflammation and maintains physiological balance through a negative feedback regulation mechanism. However, excessive, overactive, and long-term inflammation responses play a crucial part in the pathological mechanisms of inflammatory illnesses, such as atherosclerosis, type 2 diabetes mellitus, and rheumatoid arthritis (Lee and Weinblatt, 2001; Wolf and Ley, 2019; Ying et al., 2020). Macrophages, an important type of cells involved in natural immunity, exert a prominent part in generating numerous inflammatory mediators, including nitric oxide (NO), prostaglandin E_2_ (PGE_2_), interleukin-1β (IL-1β), IL-6, and tumor necrosis factor-α (TNF-α). Reduced levels of inflammatory mediators are beneficial and critical for treatment. Relevant studies have shown that botanical components of plants have outstanding pharmacologic potentials for use in inflammation therapy (Tasneem et al., 2019).

Lipopolysaccharide (LPS), a biological composition of the cell wall of gram-negative bacteria, could activate macrophages, which are associated with an uncontrolled secretion of inflammatory mediators, to further elicit infection even septic shock (He et al., 2020). Toll-like receptor 4 (TLR4) and myeloid differentiation factor 2 (MD2) form a heterodimer that specifically recognizes LPS. Five lipid chains of LPS submerge into the hydrophobic cavity of MD2, whereas the sixth chain extrudes from the MD2 hydrophobic bag and establishes interactions with TLR4, inducing the formation of an m-shaped multimer composed of two copies of the TLR4-MD2-LPS complex arranged symmetrically (Park et al., 2009; Billod et al., 2016). The effector protein is subsequently recruited to the intracellular region to further cause a series of cascade reactions, ultimately promoting the activation of nuclear factor-κB (NF-κB) and mitogen-activated protein kinases (MAPK) signaling (Ostareck and Ostareck-Lederer, 2019). Thus, the generation of inflammatory factors is accelerated. Several TLR4 signaling inhibitors have been utilized to treat inflammatory disorders in preclinical and clinical studies (Romerio and Peri, 2020).

*Panax ginseng* is a famous traditional herbal plant distributed in Asia and North America for thousands of years. *Panax ginseng* possesses various pharmacological characteristics, such as anti-inflammation, antioxidation, anticancer, antifatigue, and antiaging, and has been widely utilized in the treatment of numerous human disorders, such as inflammatory diseases, neurodegenerative diseases, and cancers (Radad et al., 2006; Iqbal and Rhee, 2020; Liu et al., 2020). Ginsenoside Rb3 (GRb3) is a main active panaxadiol of *Panax ginseng*. Previous studies have shown that GRb3 inhibited reactive oxygen species (ROS) production and NADPH oxidases in arterial rings from hypertensive rats. Moreover, GRb3 blocked angiotensin II-induced reduction of NO and phosphorylation of endothelial NOS in human umbilical vein endothelial cells and suppressed oxidative stress in renal arteries of hypertensive patients or normotensive subjects (Wang Y. et al., 2014). Chen reported that GRb3 exerted cardio-protective effects by improving energy metabolism and inhibiting myocardial apoptosis through the activation of PPARα signaling (Chen et al., 2019). Additionally, the anti-inflammation effect of GRb3 was detected on *Porphyromonas gingivalis* LPS-stimulated human periodontal ligament cells and periodontitis rats for the first time by Sun et al. (2020). It demonstrated that GRb3 ameliorated LPS-induced inflammation in human periodontal ligament cells and periodontitis rats by inhibiting MAPK/AKT/NF-κB signaling. Interestingly, it also demonstrated that GRb3 markedly decreased TLR2 mRNA expression, whereas it had little effect on TLR4 mRNA levels in LPS-stimulated human periodontal ligament cells.

In the present study, we explored the anti-inflammatory properties of GRb3 in macrophages using an LPS-mediated inflammation model. We determined whether GRb3 exerted anti-inflammatory effects by suppressing the TLR4 signaling pathway. To the best of our knowledge, this is the first report to show that GRb3 can potently inhibit LPS-induced inflammation through direct inhibition of TLR4 signaling in macrophages.

## Materials and Methods

### Materials

GRb3 and ginsenoside Rg3 (GRg3) were purchased from Chengdu Must Bio-Technology (Sichuan, China). LPS (*Escherichia coli*, O55:B5) and resatorvid (TAK242) were obtained from Sigma Aldrich (St. Louis, MO, United States). Primary antibodies targeting iNOS, COX2, p-p65 (Ser536), p65, p-IκBα (Ser32/36), p-JNK (Thr183/Tyr185), JNK, p-p38 (Thr180/Tyr182), p38, p-ERK (Thr202/Tyr204), ERK, and GAPDH were obtained from CST (Beverly, MA, United States). Anti-TLR4 antibody was commercially obtained from Abcam (Cambridge, MA, United States). Recombinant Human TLR4/MD2 Complex Protein was purchased from R&D Systems (Minneapolis, MN, United States). Other reagents were obtained commercially.

### Cell Culture and Viability

Murine macrophage cells (RAW264.7 cells) and human mononuclear cells (THP1 cells) were obtained from the Biochemistry Cell Bank (Chinese Academy of Sciences, Shanghai, China). Cells were cultured in DMEM (Gibco, Grand Island, NY, United States) containing 10% FBS, 100 units/ml penicillin, and 100 μg/ml streptomycin (Gibco) in an incubator at 37°C. THP1 macrophages were subjected to monocytic differentiation by incubation with phorbol 12-myristate-13-acetate (Sigma Aldrich, 100 nM) solution overnight. MTT reagents were utilized to measure cell viability. Macrophages were inoculated in a 96-well plate (1 × 10^4^ cells/well) overnight and incubated with the test compounds for 24 h. Cell activity assessment was performed in accordance with standard protocols.

### Lentivirus Construction and Transfection

TLR4 and negative control lentiviruses encoding green fluorescent protein (GFP) were constructed by Vigene Biosciences (Shandong, China). RAW264.7 cells were cultured in a 96- or 6-well plate. Lentivirus was utilized to transfect RAW264.7 cells at a multiplicity of infection (MOI) of 100 when cells grew to 20%–30% confluence. Subsequently, the fresh completed medium was replaced 8 h after transfection. The cells were cultivated for another 72 h, and the corresponding treatment was carried out.

### Determination of NO, PGE_2_, IL-1β, IL-6, and TNF-α

Cells were inoculated in a 6-well plate (5 × 10^5^ cells/well) overnight and treated with LPS (0.1 μg/ml) for 22 h. To detect NO levels by Griess assay, culture supernatant (50 μl) was extracted and mixed with Griess A reagent (50 μl) and Griess B reagent (50 μl) (Beyotime, Shanghai, China). A microplate reader was adopted to determine the value at 540 nm. Enzyme-linked immunosorbent assay (ELISA) kit (Reddot Biotech, British Columbia, Canada) was purchased to measure PGE_2_ levels according to the product procedures. Similarly, the levels of IL-1β, IL-6, and TNF-α were measured by mouse corresponding ELISA kits (Dakewe, Shenzhen, China) in accordance with standard protocols.

### Quantitative Reverse Transcription Polymerase Chain Reaction

Macrophages were cultivated with LPS for 6 h with or without test compounds, and the mRNA levels of inflammatory mediators were performed by quantitative reverse transcription polymerase chain reaction (qRT-PCR). The total RNA of cells was extracted by TRIzol (Accurate Biology, Hunan, China), with subsequent cDNA synthesis by Evo M-MLV RT kit with gDNA clean (Accurate Biology, AG11711) according to the manufacturers’ instructions. PCR reaction was conducted using SYBR Green Premix Pro Taq HS qPCR Kit (Accurate Biology, AG11701) by LightCycler PCR system. The mRNA levels were measured by the 2^−ΔΔCt^ method, with GAPDH as an internal normalization. Table 1 displays the primers of inflammatory mediators utilized in PCR.

**TABLE 1:** The primer sequences used for qRT-PCR.

Gene		Primer sequence 5′-3′
iNOS	Forward	5′-ACA​ACA​GGA​ACC​TAC​CAG​CTC​A-3′
	Reverse	5′-GAT​GTT​GTA​GCG​CTG​TGT​GTC​A-3′
COX2	Forward	5′-CCA​GAT​GCT​ATC​TTT​GGG​GAG​AC-3′
	Reverse	5′-CTT​GCA​TTG​ATG​GTG​GCT​G-3′
IL-1β	Forward	5′-TCA​AAT​CTC​GCA​GCA​GCA​CAT​C-3′
	Reverse	5′-CGT​CAC​ACA​CCA​GCA​GGT​TAT​C-3′
IL-6	Forward	5′-CCC​CAA​TTT​CCA​ATG​CTC​TCC-3′
	Reverse	5′-CGC​ACT​AGG​TTT​GCC​GAG​TA-3′
TNF-α	Forward	5′-TGG​AAC​TGG​CAG​AAG​AGG​CAC-3′
	Reverse	5′-AGG​GTC​TGG​GCC​ATA​GAA​CTG​A-3′
TLR4	Forward	5′-GCC​TTT​CAG​GGA​ATT​AAG​CTC​C-3′
	Reverse	5′-GAT​CAA​CCG​ATG​GAC​GTG​TAA​A-3′
GAPDH	Forward	5′-AGG​TCG​GTG​TGA​ACG​GAT​TTG-3′
	Reverse	5′-TGT​AGA​CCA​TGT​AGT​TGA​GGT​CA-3′

### Western Blot

For western blot (WB) experiment, protein samples were dissolved with RIPA containing protease and phosphatase inhibitor. BCA Protein Assay Kit (Beyotime) was utilized to measure the protein concentration. Protein samples (approximately 15 µg) were subjected to 10% SDS-PAGE, transferred to PVDF membranes (Millipore, MA, United States), and blocked with 5% skim milk for 2 h. The membranes were reacted with proper primary antibodies at 4°C overnight. Afterward, the corresponding IgG antibody (CST) was adopted for 2 h. Finally, an ECL kit (Affinity, Cincinnati, OH, United States) was adopted to visualize protein bands. Image J was performed to quantify the protein density.

### Immunofluorescence

To test p65 nucleus translocation by immunofluorescence (IF), RAW264.7 macrophages were seeded on confocal dishes overnight and incubated with LPS in the presence or absence of GRb3 for 1 h. After washing with PBS for 10 min, paraformaldehyde was employed to immobilize cells, followed by blocking cells with goat serum containing 0.1% Triton. Then, macrophages reacted with p65 antibody overnight, which was diluted at 1:400 with 5% goat serum solute. After incubation with Alexa Flour 568-combined IgG (Thermo Fisher, #A-21069) for 2 h, cells were subjected to DAPI staining (Beyotime) for 5 min. In addition, to detect whether GRb3 blunted LPS binding to the cell membranes, Alexa Fluor 488-conjugated LPS (LPS488, Sigma Aldrich, 10 μg/ml) was exposed to macrophages for 12 h. PBS was adopted to rinse cells three times, with DAPI staining for 10 min. Subsequently, a confocal microscope (CarlZeiss, Germany) was employed to determine p65 nucleus transfer and LPS uptake to cytomembranes.

### Molecular Docking and Molecular Dynamics

Autodock Vina (Scripps Research Institute, United States) was used to perform molecular docking for testing the integrating mechanisms of the TLR4/MD2 complex (PDB ID: 3FXI) with GRb3 (CID: 6440398). The water molecule and binding ligand were eliminated to prepare the molecular docking simulated structure of the TLR4/MD2 complex. YASARA was utilized to execute molecular dynamics (MD) simulation and minimize ligand energy. Herein, the ligand-combining regions of TLR4/MD2 were considered as the inhibitor-combining regions. The best model for MD simulation was regarded as a star-shaped structure. AMBER 03 forcefield was utilized to perform all simulations. NaCl (0.9%) was put at the dodecahedron case, served as a solvent for the ligand/receptor complex. The initialization of simulation annealing was 298K, according to the past practice, with speed reducing by 0.9 every 10 steps. Additionally, recalibration and adjustment of the velocities were conducted every 100 simulation steps. After minimizing energy, we adjusted the temperature for effect minimization of temperature control. Eventually, 100 ns MD simulation was performed at 2 fs, with the storage of complex coordinates in an interval of 10 ps.

### Surface Plasmon Resonance Imaging

Kx5 S instrument (Plexera, United States) was employed to perform surface plasmon resonance imaging (SPRi) analysis for probing the integrating mechanisms of TLR4/MD2 complex with GRb3. The GRb3 samples were prepared with PBS at 10, 40, and 100 μM concentrations. The typical binding curve of GRb3 to TLR4/MD2 complex comprised association for 300 s, dissociation for 300 s, and reconstruction for 200 s. All binding signal changes were transformed to standard refractive units (RU) *via* the calibration of each spot (Zhao et al., 2017). Finally, commercial SPRi analysis software (Plexera SPR Data Analysis Module, Plexera, United States) was used for the collection and analysis of the binding data.

### Statistical Analysis

Data were presented as mean ± SD after analysis by SPSS 21.0 (IBM Corporation, Chicago, United States). One-way ANOVA was utilized to determine the difference of quantitative data among multiple groups, while Student’s t-test was performed to examine the difference between the two groups. *p* < 0.05 and *p* < 0.01 were considered statistically significant.

## Results

### GRb3 Inhibited NO and PGE_2_ Generation by Inhibiting iNOS and COX2 Expression in LPS-Stimulated Macrophages

**FIGURE 1:**
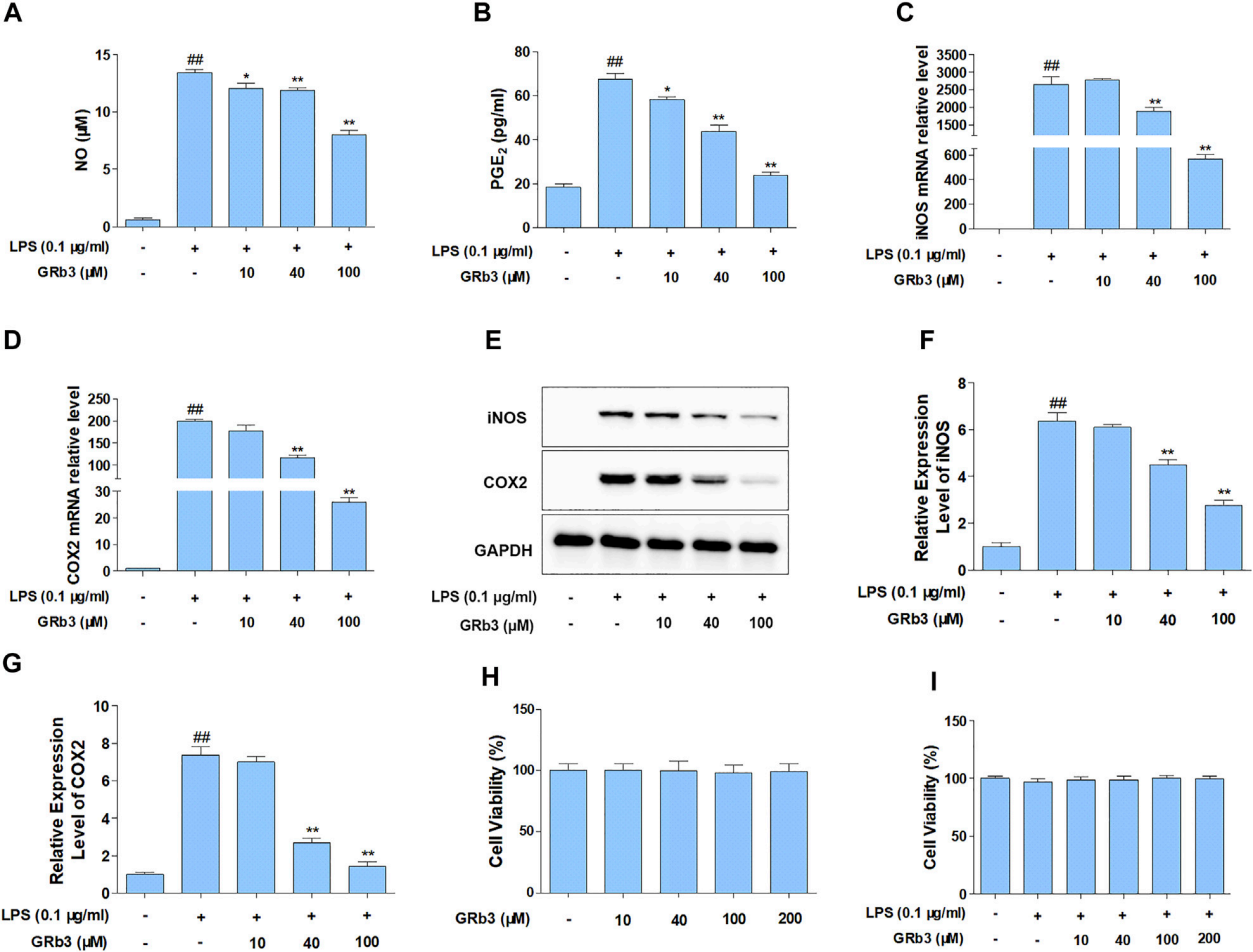
GRb3 inhibited NO and PGE_2_ generation by inhibiting iNOS and COX2 expression in LPS-treated RAW264.7 cells. RAW264.7 cells were first incubated with GRb3 of indicated concentrations (10, 40, and 100 µM) for 2 h and further co-treated with LPS (0.1 μg/ml) for 22 h, followed by detection of NO **(A)** and PGE_2_ **(B)** expression by Griess reagent and ELISA assay, respectively (*n* = 3). In a parallel assay, we extracted total RNA under LPS administration for 6 h, followed by qRT-PCR to determine the mRNA expression of iNOS **(C)** and COX2 **(D)** (*n* = 3). GAPDH was taken as a reference. **(E)** RAW264.7 macrophages were first incubated with GRb3 (10, 40, and 100 µM) for 2 h and subsequently co-treated with LPS (0.1 μg/ml) for 22 h, followed by WB to measure iNOS and COX2 protein expression. Relative expression of iNOS **(F)** and COX2 **(G)** was subjected to the normalization of GAPDH (*n* = 3). **(H)** Cell viability of GRb3 in RAW264.7 cells (*n* = 6). **(I)** Cell viability of GRb3 in LPS-treated RAW264.7 cells (*n* = 6). Data were displayed as mean ± SD. ^#^ *p* < 0.05, ^##^ *p* < 0.01 versus the control group. *p < 0.05, **p < 0.01 versus the LPS-treated group.

For detecting the suppressive roles of GRb3 on the generation of NO and PGE_2_, RAW264.7 cells were pre-treated with GRb3 (10, 40, and 100 μM) for 2 h, followed by co-treatment with LPS (0.1 μg/ml) for 22 h. As shown in Figures 1A,B, treatment with LPS alone obviously increased NO and PGE_2_ production compared with the control group. However, GRb3 administration decreased NO and PGE_2_ secretion dose-dependently in RAW264.7 cells. Since inducible nitric oxide synthases (iNOS) and cyclooxygenase 2 (COX2) could modulate the expression of NO and PGE_2_, respectively, we further examined whether GRb3 decreased iNOS and COX2 expression. As a result, GRb3 dose-dependently decreased the mRNA and protein levels of iNOS and COX2 (Figures 1C–G). In addition, we supplemented a TLR4 selective inhibitor (TAK242) as a positive control in LPS-induced inflammation. From our results, the effect of GRb3 (100 μM) was equivalent to that of 100 nM TAK242 on the protein and mRNA levels of iNOS and COX2 (Supplementary Figures S1A–D). Consistently, iNOS and COX2 expression in THP1 cells were significantly suppressed by GRb3 and TAK242 (Supplementary Figures S1E–H). Furthermore, to investigate the toxicity of GRb3, cells were incubated with GRb3 (10, 40, 100, and 200 μM) for 24 h. MTT assay showed no significant difference in cell viability between GRb3 and control groups, suggesting that GRb3 had no cytotoxicity to RAW264.7 cells (Figure 1H). Meanwhile, compared with the control group, 0.1 μg/ml LPS failed to impair cell viability, and the survival of cells treated with GRb3 was similar to that of cells in the LPS group (Figure 1I).

### GRb3 Suppressed IL-1β, IL-6, and TNF-α Expression in LPS-Treated Macrophages

**FIGURE 2:**
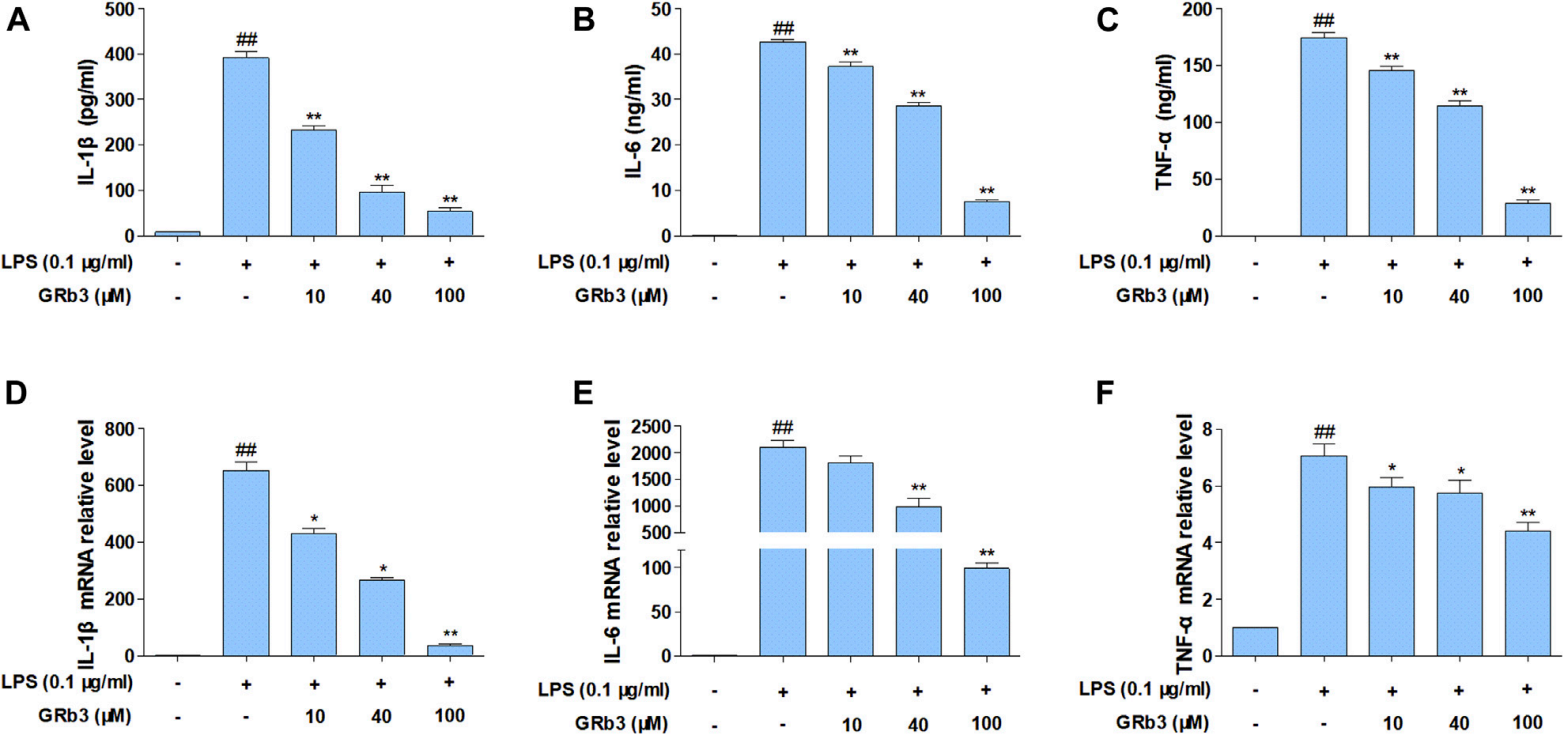
GRb3 inhibited IL-1β, IL-6, and TNF-α expression in RAW264.7 cells. RAW264.7 cells were incubated with GRb3 of indicated dose (10, 40, and 100 µM) for 2 h and further co-treated with LPS (0.1 μg/ml) for 22 h, followed by ELISA to measure the production of IL-1β **(A)**, IL-6 **(B)**, and TNF-α **(C)** in the culture supernatant. In a parallel assay, we isolated total RNA with LPS treatment for 6 h, followed qRT-PCR to quantify the mRNA expression of IL-1β **(D)**, IL-6 **(E)**, and TNF-α **(F)**. GAPDH was utilized as an internal reference. Data were presented as mean ± SD of three independent experiments. ^#^ *p* < 0.05, ^##^ *p* < 0.01 versus the control group. *p < 0.05, **p < 0.01 versus the LPS-treated group.

We subsequently examined the functions of GRb3 in pro-inflammatory cytokines expression. Cells were first reacted with GRb3 for 2 h and then co-treated with LPS for 22 h. Consequently, the secretion of IL-1β, IL-6, and TNF-α was upregulated by LPS administration alone. However, GRb3 dose-dependently decreased IL-1β, IL-6, and TNF-α secretion (Figures 2A–C). In addition, we further detected the mRNA levels of IL-1β, IL-6, and TNF-α in macrophages. As displayed in Figures 2D–F, GRb3 treatment significantly attenuated LPS-mediated upregulated mRNA levels of IL-1β, IL-6, and TNF-α dose-dependently in RAW264.7 cells. Moreover, as shown in Supplementary Figures S1I–K, the effect of 100 μM GRb3 was equal to that of TAK242 on the mRNA expression of these inflammatory cytokines. Consistent with RAW264.7 cells findings, GRb3 also blunted the mRNA levels of IL-1β, IL-6, and TNF-α in THP1 cells (Supplementary Figures S1L–N).

### GRb3 Suppressed NF-κB and MAPK Activation in LPS-Treated Macrophages

**FIGURE 3:**
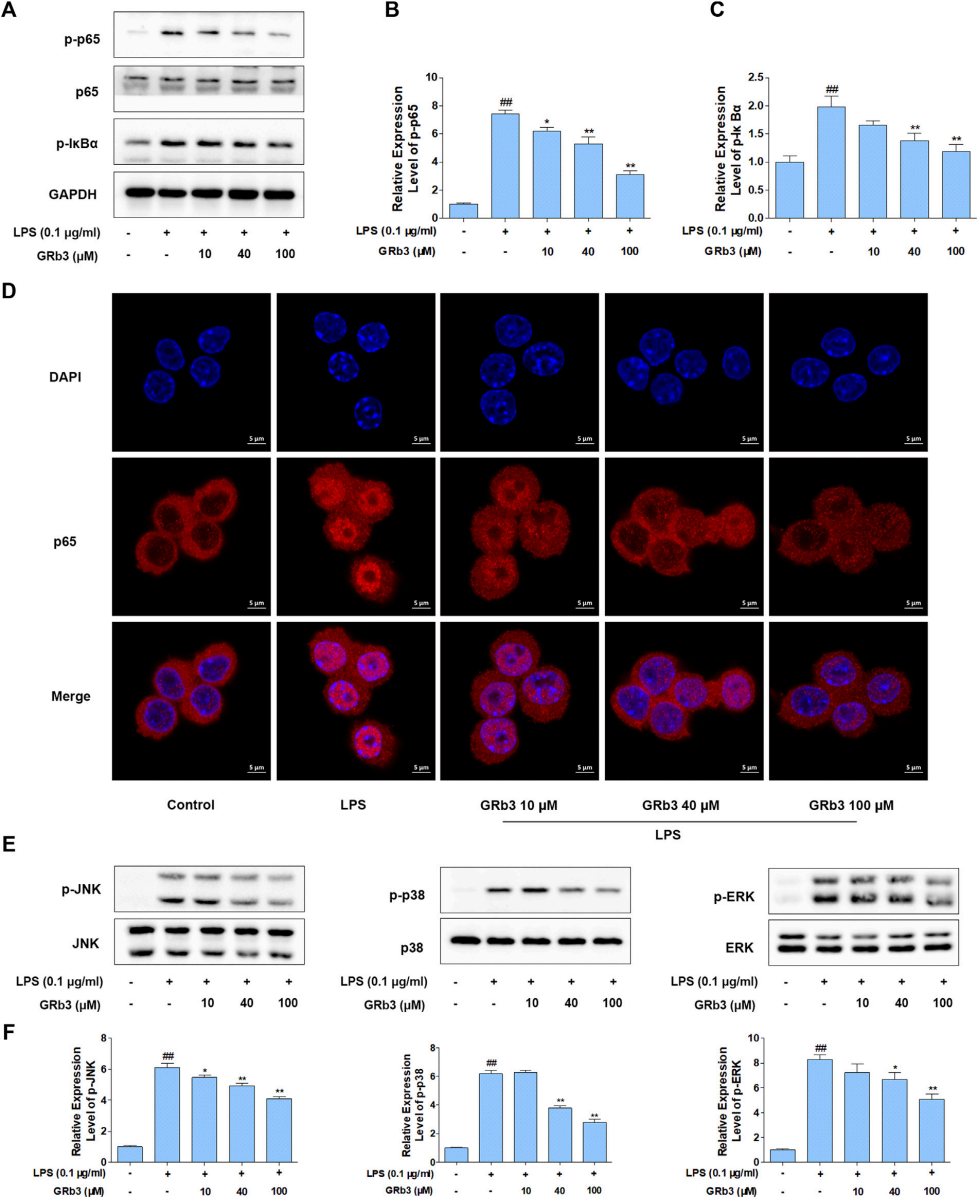
GRb3 inhibited NF-κB and MAPK activation in LPS-treated RAW264.7 cells. **(A)** After incubation with GRb3 (10, 40, and 100 µM) for 24 h, RAW264.7 macrophages were subsequently co-treated with LPS (0.1 μg/ml) for 1 h, followed by WB to test p-p65, p65, and p-IκBα expression. **(B)** The relative expression level of p-p65 was normalized with p65. **(C)** The relative expression level of p-IκBα was normalized with GAPDH. **(D)** IF was employed to measure the nuclear translocation of NF-κB (p65). The red regions (p65) represented the distribution of p65, and the blue regions (DAPI) represented cellular nuclei. **(E)** Effect of GRb3 on phosphorylation of JNK, p38, and ERK, and their relative expression levels were normalized to JNK, p38, and ERK **(F)**. Data were presented as mean ± SD of three independent experiments. ^#^ *p* < 0.05, ^##^ *p* < 0.01 versus the control group. *p < 0.05, **p < 0.01 versus the LPS-treated group.

IκBα phosphorylation and subsequent p65 nucleus migration activate NF-κB signaling, which is associated with the expression of inflammatory mediators (Ferreiro and Komives, 2010; Mitchell and Carmody, 2018). In addition, post-translational modification of NF-κB has a distinguished efficacy in activating NF-κB, of which the p65 phosphorylation is the most important post-translational modification (Huang et al., 2010). Therefore, we examined the possible inhibitory roles of GRb3 in p65 and IκBα phosphorylation levels and p65 nuclear translocation in LPS-treated macrophages. We observed that LPS treatment led to the hyper-phosphorylation status of p65 and IκBα in RAW264.7 macrophages. Nevertheless, GRb3 administration decreased the phosphorylation of p65 as well as IκBα (Figures 3A–C). IF was subsequently conducted to determine the roles of GRb3 in p65 nucleus translocation. Consequently, LPS induced p65 subunit nuclear accumulation. However, treatment with GRb3 alleviated LPS-stimulated p65 nuclear accumulation (Figure 3D). These outcomes suggested that GRb3 can inhibit NF-κB activation by abolishing p65 nuclear accumulation through suppression of IκBα phosphorylation. Considering the reported roles of MAPK in inflammatory mediator generation (Kaminska, 2005), GRb3’s possible effect on phosphorylation of JNK, p38, and ERK was evaluated. WB illustrated that hyper-phosphorylation of JNK, p38, and ERK was conspicuous in the LPS group, but it was blocked by GRb3 treatment without changing the total protein (Figures 3E,F). Then, we further evaluated the effects of TAK242 on LPS-induced NF-κB/MAPK signaling activation. We found that TAK242 significantly reduced the phosphorylation levels of IκBα, p65, JNK, p38, and ERK (Supplementary Figures S2A–D). Similarly, the expression of p-IκBα, p-p65, p-JNK, p-p38, and p-ERK in THP1 cells was significantly suppressed by GRb3 and TAK242 compared with the LPS group (Supplementary Figures S2E–H).

### Molecular Docking and MD Simulation

**FIGURE 4:**
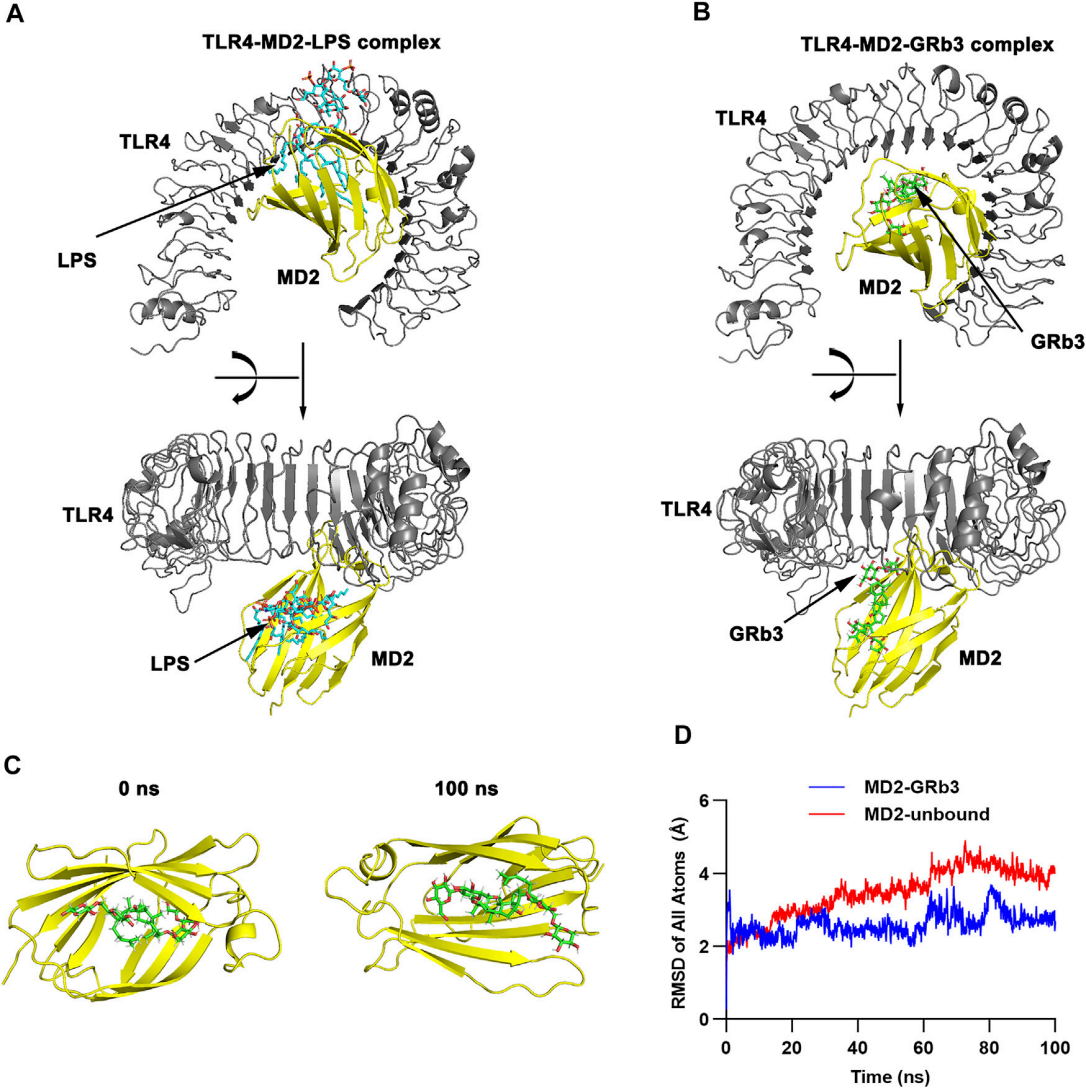
Molecular docking and MD simulation. **(A)** 3D modeling of TLR4-MD2-LPS complex. **(B)** 3D modeling of TLR4-MD2-GRb3 complex. **(C)** Visualization modeling of MD2-GRb3 at 0 and 100 ns. **(D)** RMSD of TLR4-MD2-GRb3 complexes (blue line) and TLR4-MD2-unbound (red line).

TLR4, as a specific receptor of LPS, can activate NF-κB and MAPK signaling pathway and modulate the expression of the inflammatory cytokines (Ostareck and Ostareck-Lederer, 2019). To explore whether GRb3 played a protective role in LPS-treated macrophages by targeting TLR4 signaling, the binding mechanism of TLR4/MD2 with GRb3 was detected by molecular docking. Figures 4A,B displays the three-dimensional (3D) conformation of TLR4-MD2-LPS complex and TLR4-MD2-GRb3 complex. The bond energy of TLR4-MD2-GRb3 was found to be −8.79 kcal/mol, which suggested a strong combining capacity. Figure 4C demonstrates the visual conformations of the MD2-GRb3 complex at 0 and 100 ns, where GRb3 appeared closely in the MD2 binding center site until the termination of MD simulation. Additionally, according to the trajectories of the root mean square deviation (RMSD) of the TLR4-MD2-GRb3 complex (Figure 4D, blue line) and unbound MD2 (MD2-free) (Figure 4D, red line), the RMSD of MD2-GRb3 steadily fluctuated at 2–3 Å. Moreover, we have supplemented the docking amino acid residues of MD2 binding to GRb3. Supplementary Figure S3 displays that 22 hydrogen bonds comprised GRb3 with Lys122, Phe126, Phe121, Ile80, Cys133, Lys132, Tyr131, Ile153, Phe151, Arg90, Lys91, Cys95, Tyr102, Val93, Arg96, Ile94, Ile124, Arg264, Glu92, Asp101, Asn339 and Asp100 of MD2. These consequences indicated that the interaction between TLR4/MD2 and GRb3 was stable, and GRb3 might directly target TLR4 signaling to regulate the inflammation induced by LPS in macrophages.

### GRb3 Directly Targeted TLR4 in LPS-Treated Macrophages

**FIGURE 5:**
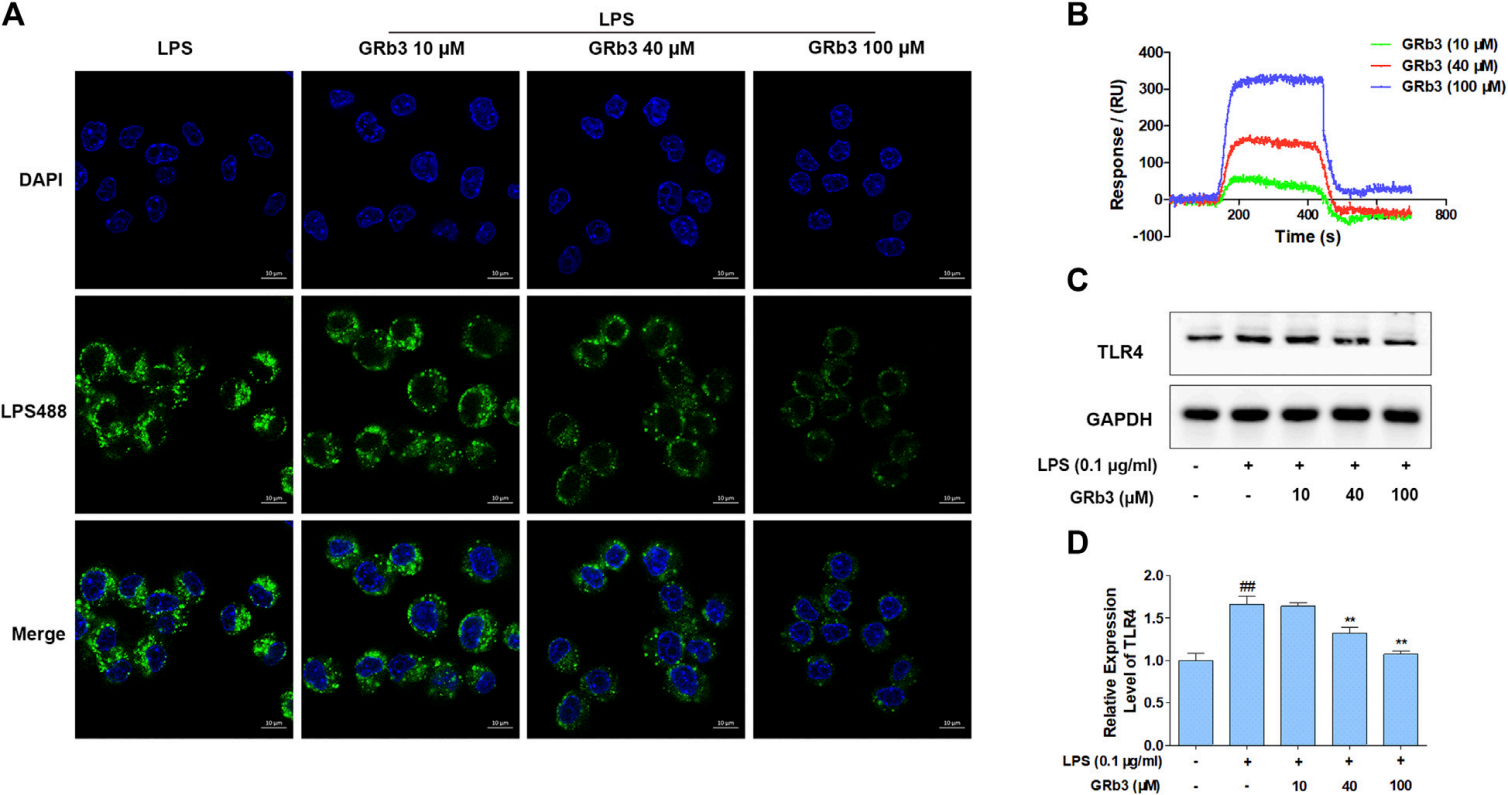
GRb3 directly targeted TLR4. **(A)** The binding of LPS488 with cell membranes was detected by IF after GRb3 (10, 40, and 100 µM) co-treatment with LPS488 simultaneously for 12 h. In the representative images, the green region represented LPS488, whereas the blue region represented the cells’ nuclei stained with DAPI. **(B)** SPRi was performed to investigate the interactions between GRb3 and TLR4/MD2 complex and test binding affinity values. **(C)** After incubation with GRb3 (10, 40, and 100 µM) for 24 h, RAW264.7 macrophages were co-treated with LPS (0.1 μg/ml) for 1 h, followed by WB to test TLR4 expression. **(D)** The relative expression level of TLR4 was normalized with GAPDH. Data were presented as mean ± SD of three independent experiments. ^#^ *p* < 0.05, ^##^ *p* < 0.01 versus the control group. *p < 0.05, **p < 0.01 versus. the LPS-treated group.

IF, SPRi, and WB were further performed to detect the pharmacological action of GRb3 on TLR4. Consequently, treatment with GRb3 blunted the fluorescence of LPS488 attaching to the cytomembranes in a concentration-dependent manner (Figure 5A). SPRi demonstrated that GRb3 could bind to TLR4/MD2 complex instantly (Figure 5B). The equilibrium dissociation constant (K_D_) value was 6.23 × 10^–9^ M, indicating a great binding capacity. Moreover, as shown in Figures 5C,D, TLR4 protein level was upregulated by LPS. However, GRb3 significantly decreased the expression of TLR4 in macrophages. The outcomes confirmed that GRb3 targeted TLR4 directly.

### Suppression of TLR4 Did not Increase Suppressive Roles of GRb3 in RAW264.7 Macrophages

**FIGURE 6:**
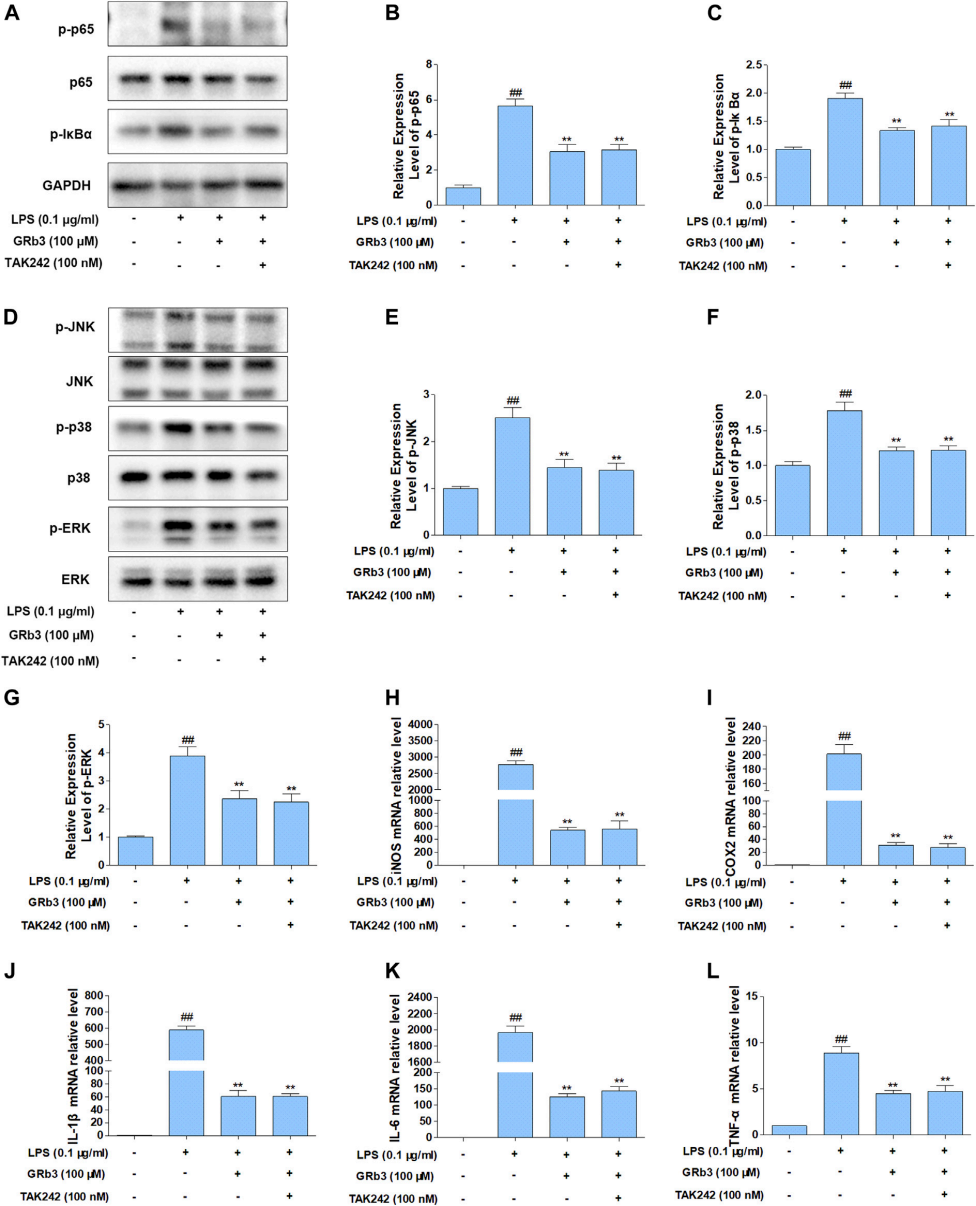
Suppression of TLR4 did not increase the suppressive impact of GRb3 in RAW264.7 macrophages. **(A)** After incubation with selective TLR4 inhibitor (TAK242, 100 nM) for 1 h, RAW264.7 cells were co-incubated with GRb3 (100 μM) for 22 h, subsequently treated with LPS (0.1 μg/ml) for 1 h, followed by WB to test p-p65, p65, and p-IκBα expression. **(B)** Relative expression of p-p65 was normalized with p65. **(C)** Relative expression of p-IκBα was normalized with GAPDH. **(D)** WB was adopted to measure the levels of p-JNK, p-p38, and p-ERK, and their relative expression levels were normalized to JNK, p38, and ERK **(E–G)**. In a parallel assay, total RNA were extracted after LPS treatment for 6 h, and qRT-PCR was performed to quantify the mRNA expression of iNOS **(H)**, COX2 **(I)**, IL-1β **(J)**, IL-6 **(K)**, TNF-α **(L)**. GAPDH was taken as the loading control. Data were presented as mean ± SD of three independent experiments. ^#^ *p* < 0.05, ^##^ *p* < 0.01 versus the control group. *p < 0.05, **p < 0.01 versus the LPS-treated group.

A selective TLR4 inhibitor, TAK242, was applied in the next experiments to assess whether TLR4 mediated the suppressive influences of GRb3 on NF-κB/MAPK signaling. RAW264.7 macrophages were pre-incubated with 100 nM TAK242 for 1 h and then co-incubated with 100 μM GRb3 for another 22 h. Finally, they were incubated with 0.1 μg/ml LPS for 1 h, followed by WB to detect the expression of p-p65, p-IκBα, p-JNK, p-p38, and p-ERK. As shown in Figures 6A–C, the expression levels of p-p65 and p-IκBα were decreased by GRb3. However, co-treatment with GRb3 and TAK242 failed to enhance the inhibitory effect of GRb3 on NF-κB compared with the GRb3 treatment alone. Consistently, phosphorylation levels of JNK, p38, and ERK were not further significantly reduced in the GRb3 and TAK242 co-treated group compared with cells cultivated by GRb3 (Figures 6D–G). The above results suggested that GRb3’s suppressive impacts on NF-κB/MAPK signal were at least partially caused by the inhibition of TLR4. To further detect the involvement of TLR4 in the inhibitory effects of GRb3 on inflammatory mediators, qPCR was used to detect iNOS, COX2, IL-1β, IL-6, and TNF-α mRNA levels. As a result, co-treatment with GRb3 and TAK242 did not enhance the inhibitory influences of iNOS, COX2, IL-1β, IL-6, and TNF-α in comparison with GRb3 treatment after LPS exposure (Figure 6H–L). The inhibitory impacts of GRb3 on these inflammatory mediators were at least partially caused by the suppression of TLR4 activation.

### Overexpression of TLR4 Significantly Reversed the Suppressive Roles of GRb3 on NF-κB/MAPK Signal and Inflammatory Mediators

**FIGURE 7:**
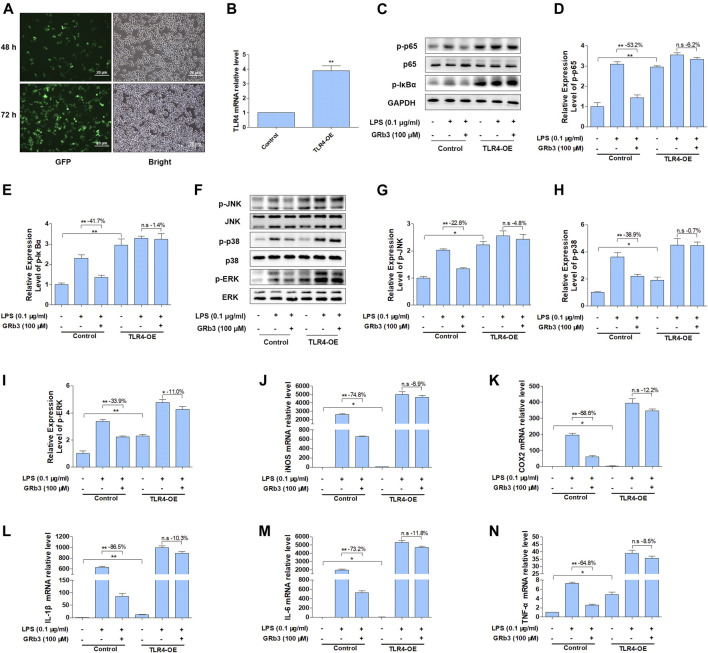
Overexpression of TLR4 reversed the suppressive impact of GRb3 on NF-κB/MAPK signal and inflammatory mediators. RAW264.7 cells were transfected with TLR4 overexpression or control lentivirus for 72 h and then treated with LPS or GRb3. **(A)** GFP fluorescence and bright field images in cells transduced with TLR4 lentivirus 48 and 72 h after transfection (*n* = 3). **(B)** The mRNA levels of TLR4 in control and TLR4 lentivirus interfered groups (*n* = 3). **(C–E)** WB was adopted to measure p-p65 and p-IκBα, and their relative expression levels were normalized to p65 and GAPDH (*n* = 3). **(F–I)** WB was utilized to detect p-JNK, p-p38, and p-ERK and their relative expression levels (*n* = 3). In a parallel assay, total RNA were extracted after LPS treatment for 6 h, and the mRNA expression levels of iNOS **(J)**, COX2 **(K)**, IL-1β **(L)**, IL-6 **(M)**, and TNF-α **(N)** were quantified by qRT-PCR (*n* = 4). GAPDH was taken as the loading control. Data were presented as mean ± SD. *p < 0.05, **p < 0.01, n. s, no significant differences.

To further evaluate whether GRb3 inhibited LPS-induced expression of inflammatory mediators by inhibiting TLR4/NF-κB/MAPK signaling pathways activation, we overexpressed the TLR4 gene in RAW264.7 cells using TLR4 lentivirus. RAW264.7 cells were transfected with TLR4 or control lentivirus for 72 h and then treated with LPS or GRb3. Figure 7A displays that GFP fluorescence obviously could be observed in cells transduced with TLR4 lentivirus 48 and 72 h after transfection. QPCR result demonstrated that TLR4 mRNA level was significantly increased in TLR4 lentivirus interfered group compared with the control group (Figure 7B), indicating that TLR4 lentivirus was successfully transfected. WB analysis demonstrated that p-p65 and p-IκBα were markedly reduced by GRb3 in cells interfered with control lentivirus. However, overexpression of TLR4 reversed the inhibitory effect of GRb3 on p-p65 and p-IκBα (Figures 7C–E). Similarly, the repressive roles on the phosphorylation levels of JNK, p38, and ERK were partially abolished when TLR4 was highly expressed (Figures 7F–I). These consequences indicated that GRb3 suppressed NF-κB/MAPK pathway activation in a TLR4-dependent mode. We further determined the association between the suppressive impacts of GRb3 on inflammatory factors and inhibitory TLR4 activation. As presented in Figures 7J–N, the overexpression of TLR4 largely eliminated the repressive effect of GRb3 on these inflammatory mediators. Taken together, these results suggested that TLR4 was the main pharmacological target of GRb3 for NF-κB/MAPK signal suppression and its anti-inflammatory effects.

## Discussion

Inflammation is a complex pathological process and the basic pathogenesis in multiple inflammation-related disorders, including rheumatoid arthritis, atherosclerosis, diabetes, and cancer. Ginsenosides are the major active ingredients of ginseng and are extracted from the roots, stems, and leaves of ginseng. According to the differences in the position and quantity of sugar moiety, ginsenosides are divided into three types (Kim et al., 2007; Wang L. et al., 2014; Shin and Oh, 2014): panaxadiol group (ginsenosides Rb1, Rb2, Rb3, Rc, Rd, Rg3, and Rh2), panaxatriol group (ginsenosides Re, Rg1, Rg2, and Rh1), and oleanolic acid group (ginsenoside Ro). Each ginsenoside plays a different pharmacological role as shown in bioactivity studies such as anticancer, anti-inflammation, antioxidation, antiaging, antifatigue, and other physiological functions (Tan et al., 2013; Li et al., 2014; Li et al., 2016; Cui et al., 2017). Notably, according to literature studies, GRb3, as a panaxadiol of *Panax ginseng*, possesses anti-inflammatory, anti-antioxidant, and immunomodulatory properties (Wang et al., 2018; Sun et al., 2019). However, the involvement of TLR4 in the suppressive activities of GRb3 on LPS-induced inflammation in macrophages is still unclear. Our present study provided a novel perspective into the underlying mechanism of the anti-inflammatory roles of GRb3 *in vitro*.

Macrophages exert a crucial role in the inflammatory responses, which are capable of secreting excessive pro-inflammatory mediators. NO is involved in the pathological mechanisms of inflammatory diseases, leading to multiple organ dysfunction in sepsis (Spiller et al., 2019). Similarly, PGE_2_ could potentiate vasodilatation, blood flow, and vascular permeability, leading to the recruitment of neutrophils from the bloodstream to infectious locations, aggravating inflammation (Kawahara et al., 2015). Thus, suppressing NO and PGE_2_ might be a beneficial therapy. We detected whether GRb3 could inhibit NO and PGE_2_ production, finding that NO and PGE_2_ were decreased by GRb3 dose-dependently. Then, we examined whether the suppressive NO and PGE_2_ production of GRb3 was regulated by iNOS and COX2, showing that GRb3 significantly inhibited the mRNA and protein expression of iNOS and COX2. Moreover, IL-1β, IL-6, and TNF-α are predominant pro-inflammatory cytokines that lead to the endotoxic shock. Consistent with the previous research (Sun et al., 2020), GRb3 significantly inhibited the production of these pro-inflammatory cytokines. The results suggested that GRb3 could inhibit LPS-mediated the expression of these inflammatory mediators.

As natural products possess an extensive spectrum of pharmacological activities against illnesses, including inflammatory diseases, they provide an infallible source for developing new drugs. Computer-aided drug design has been considered a prominent method for probing novel compounds with the desired characteristics and greatly increases the efficiency of drug discovery on a systematic, rational basis (Oglic et al., 2018). In the case of the rapid growth of drug data, molecular docking and SPRi offer a rational architecture to discover and prioritize natural products with desirable properties, promoting the transformation of these compounds into useful medicine. For example, molecular docking, one of the main tools for drug design by computers, could effectively predict the combined mechanism of receptor-ligand complex. It is broadly adopted in the development of drug studies (Chen et al., 2020). Herein, molecular docking revealed that the binding energy of the TLR4-MD2-GRb3 complex was −8.79 kcal/mol, implicating a strong binding interaction. Moreover, SPRi is a label-free, optical, and high-throughput technology that depends on light reflectivity alterations during molecular absorption on the metal surface. SPRi has been widely used to assess intermolecular interactions in a quantitative and high-throughput manner (Scarano et al., 2010). It affirmed an excellent binding capability between GRb3 and TLR4/MD2 complex. Previous studies have shown that Phe121, Phe126, and Tyr131 of MD2 play a vital part in TLR4 activity, in which alanine substitution almost eliminated LPS-induced cellular activation (Teghanemt et al., 2008). Kobayashi illustrated that Phe126 phenylalanine of MD2 induces the TLR4-MD2 polymerization, which was proved by mutations datum (Kobayashi et al., 2006). Moreover, MD2’s Cys95 and Cys105 might develop a disulfide linkage for TLR4-MD2 clustering, which are indispensable to endotoxin stimulation. Meanwhile, mutations in Cys95, Tyr102, and Cys105 residues reduced the formation of the TLR4-MD2 complex (Kawasaki et al., 2003; Re and Strominger, 2003). Mutagenesis of either Lys89-Arg90-Lys91 or Lys125-Lys125 residues abolished signaling conversion by LPS (Gruber et al., 2004). These binding sites exert a pivotal role in TLR4/MD2 activation. Consequently, we found GRb3 can bind to Phe121, Phe126, Tyr131, Cys95, Tyr102, Arg90, and Lys91 in the active site of MD2. Therefore, the binding of GRb3 and TLR4/MD2 complex is effective.

LPS is a forceful trigger of the innate immune response. TLR4 and MD2 form a heterodimer, which can recognize the common “patterns” in structurally diverse LPS molecules. The hydrophobic pocket of MD2 could accommodate most of the lipid portion of LPS, whereas the six acyl chains of LPS are left outside the pocket and interact with the ectodomain of a neighboring TLR4 molecule. Additionally, the phosphate groups of lipid A interact with positively charged amino acids of TLR4. By simultaneously binding to MD2 and the adjacent TLR4 receptor, LPS facilitates the formation of the “M” shaped dimers of TLR4/MD2 complexes, driving TLR4 activation and modulating cell signaling *via* the TLR4/NF-κB/MAPK cascades to activate pro-inflammatory mediators (Park et al., 2009; Resman et al., 2009; Płóciennikowska et al., 2015). These facts explain why GRb3 binding to TLR4 leads to the downregulation of TLR4 protein levels. Because GRb3 competitively combines with TLR4/MD2 complex and blocks LPS binding to TLR4/MD2 complexes, it could inhibit polymerization of TLR4/MD2/LPS complexes, thereby blocking TLR4 activation and downregulating TLR4 protein levels. Previous studies have displayed that the treatment with GRb3 had little effect on TLR4 mRNA expression in LPS-stimulated human periodontal ligament cells (Sun et al., 2020). The different roles of GRb3 in TLR4 signaling might be cell-type-dependent.

**FIGURE 8:**
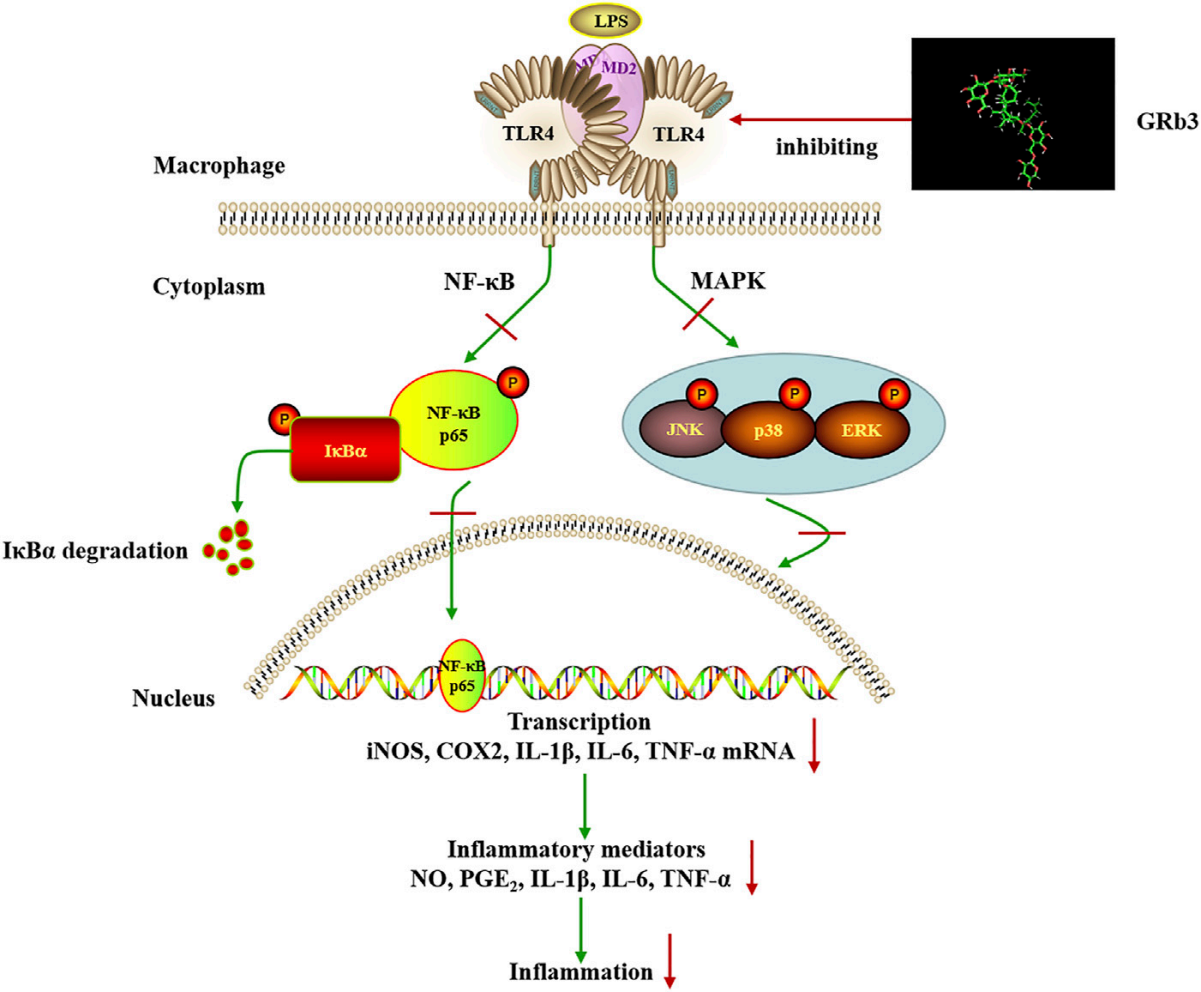
Schematic illustration of the proposed mechanism involved in the anti-inflammatory effects of GRb3 in LPS-induced macrophage.

To further confirm the involvement of TLR4 signaling in the suppressive roles of GRb3, a selective TLR4 inhibitor, TAK242, was used in the follow-up experiments. Co-treatment with GRb3 and TAK242 failed to further enhance the suppressive impacts of GRb3 on NF-κB and MAPK signaling. Additionally, the mRNA levels of inflammatory mediators were not further significantly reduced in the GRb3 and TAK242 co-treated group compared with cells cultivated by GRb3. Then, RAW264.7 cells were interfered with TLR4 lentivirus to induce TLR4 overexpression. We found that the repressive effects on the activation of NF-kB/MAPK signaling and inflammatory mediators were reversed when TLR4 was highly expressed. These findings indicated that GRb3 inhibited LPS-induced inflammation, at least in part, by inhibiting TLR4 signaling activation. As we know, multiple targets and complicated signaling pathways are essential features of natural products. In this study, we only explored a portion of the TLR4 functions and one of the pharmacology activities of GRb3 in LPS-induced inflammation. Other underlying mechanisms may be involved in GRb3’s protective effects against inflammation. Our future studies will focus on in-depth research on the pharmacology activities of GRb3 both *in vivo* and *in vitro*. Additionally, TLR4 has been demonstrated to be related to various pathological progression, including autoimmune diseases, neurodegenerative diseases, atherosclerosis, and cancer (den Dekker et al., 2010; Liu et al., 2014; Pandey et al., 2018; Leitner et al., 2019). Our results suggested that GRb3 may be capable of modulating this progression. In summary, the above outcomes revealed that GRb3 could decrease the expression of inflammatory mediators *via* a direct inhibition of TLR4/NF-κB/MAPK signaling pathways in macrophages (Figure 8).

## Data Availability

The original contributions presented in the study are included in the article/Supplementary Material. Further inquiries can be directed to the corresponding authors.

## Supplementary Material

Supplementary Figure S1 The effects of GRb3 and TAK242 on LPS-induced inflammatory mediators in RAW264.7 cells and THP1 cells. **(A)** RAW264.7 cells were incubated with 100 µM GRb3 or 100 nM TAK242 for 2 h and subsequently co-treated with LPS for 22 h. WB was used to measure iNOS and COX2 protein expression. **(B)** The relative expression of iNOS and COX2 was normalized with GAPDH. **(C, D)** The mRNA expression of iNOS and COX2 in RAW264.7 cells. **(E)** THP1 cells were incubated with 100 µM GRb3 or 100 nM TAK242 for 2 h and subsequently co-treated with LPS for 22 h. WB was used to measure iNOS and COX2 protein expression. **(F)** The relative expression levels of iNOS and COX2. **(G, H)** The mRNA expression of iNOS and COX2 in THP1 cells. **(I-K)** The mRNA expression of IL-1β, IL-6, and TNF-α in RAW264.7 cells. **(L-N)** The mRNA levels of IL-1β, IL-6, and TNF-α in THP1 cells. Data were displayed as mean ± SD (*n* = 3). ^#^ *p* < 0.05, ^##^ *p* < 0.01 versus the control group. **p* < 0.05, ***p* < 0.01 versus the LPS-treated group.

Supplementary Figure S2 The effects of GRb3 and TAK242 on TLR4/NF-κB/MAPK signaling in RAW264.7 cells and THP1 cells. **(A)** After incubation with 100 µM GRb3 or 100 nM TAK242 for 24 h, RAW264.7 cells were co-treated with LPS for 1 h, followed by WB to test TLR4, p-IκBα, and p-p65 expression. **(B)** The relative expression levels of TLR4, p-IκBα, and p-p65. **(C)** Effects of GRb3 and TAK242 on the expression of p-JNK, p-p38, and p-ERK in RAW264.7 cells. Their relative expression levels were standardized to JNK, p38, and ERK **(D)**. **(E)** THP1 cells were incubated with 100 µM GRb3 or 100 nM TAK242 for 24 h, subsequently co-treated with LPS for 1 h. WB was used to measure TLR4, p-IκBα, and p-p65 protein expression. **(F)** The relative expression levels of TLR4, p-IκBα, and p-p65 in THP1 cells. **(G)** Effects of GRb3 and TAK242 on the expression of p-JNK, p-p38, and p-ERK in THP1 cells. Their relative expression levels were standardized to JNK, p38, and ERK **(H)**. Data were displayed as mean ± SD (*n* = 3). ^#^ *p* < 0.05, ^##^ *p* < 0.01 versus the control group. **p* < 0.05, ***p* < 0.01 versus the LPS-treated group.

Supplementary Figure S3 The docking amino acid residues of MD2 to GRb3.

## Abbreviations

COX2, cyclooxygenase 2; ELISA, enzyme-linked immunosorbent assay; GRb3, ginsenoside Rb3; IF, immunofluorescence; IL-1β, interleukin-1β; IL-6, interleukin-6; iNOS, inducible nitric oxide synthases; LPS, lipopolysaccharide; MAPK, mitogen-activated protein kinases; MD, molecular dynamics; MD2, myeloid differentiation protein 2; MTT, 3-(4,5-dimethylthiazol-2-yl)-2,5-diphenyltetrazolium bromide; NF-κB, nuclear factor-κB; NO, nitric oxide; PGE_2_, prostaglandin E_2_; qRT-PCR, quantitative reverse transcription polymerase chain reaction; RA, rheumatoid arthritis; RMSD, root mean square deviation; SD, standard deviation; SPRi, Surface plasmon resonance imaging; TLR4, Toll-like receptor 4; TNF-α, tumor necrosis factor-α; WB, western blot; 3D, three-dimensional.
