# Supplementary material for: Anti-Inflammatory Effects of Ginsenoside Rb3 in LPS-Induced Macrophages Through Direct Inhibition of TLR4 Signaling Pathway
Source: Front Pharmacol. 2022 Mar 24;13:714554. doi: 10.3389/fphar.2022.714554 (PMC8987581; doi:10.3389/fphar.2022.714554)
Supplement: Supplementary file 1 [file Presentation1.pdf]

# *Supplementary Material*

# Anti-Inflammatory Effects of Ginsenoside Rb3 in LPS-Induced Macrophages Through Direct Inhibition of TLR4 Signaling Pathway

Honglin Xu[1,†] , Min Liu[2,†], Guanghong Chen[1], Yuting Wu[1,3], Lingpeng Xie[1], Xin Han[1], Guoyong Zhang[1], Zhangbin Tan[4], Wenjun Ding[4], Huijie Fan[5], Hongmei Chen[1], Bin Liu[4*] and Yingchun Zhou[1*]

*** Correspondence:** Yingchun Zhou, Email: zhychun@126.com.
Bin Liu, Email: xmhoolv@163.com.

## Supplementary Figures

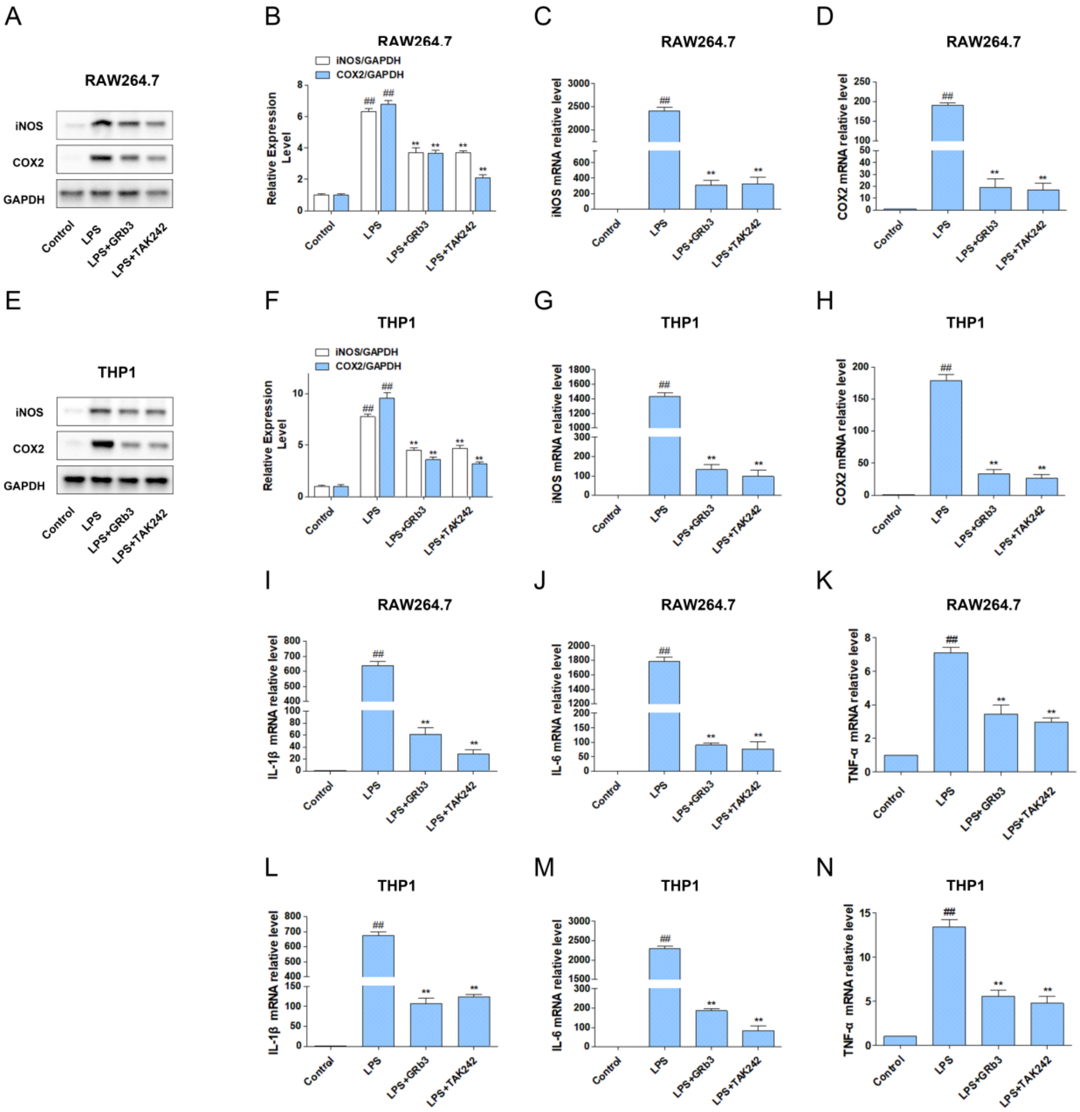

**Supplementary Figure S1. The effects of GRb3 and TAK242 on LPS-induced inflammatory mediators in RAW264.7 cells and THP1 cells.** (A) RAW264.7 cells were incubated with 100 μM GRb3 or 100 nM TAK242 for 2 h, subsequently cotreated with LPS for 22 h, WB was used to measure iNOS and COX2 protein expression. (B) The relative expression of iNOS and COX2 was normalized with GAPDH. (C, D) The mRNA expression of iNOS, COX2 in RAW264.7 cells. (E) THP1 cells were  incubated with 100 μM GRb3 or 100 nM TAK242 for 2 h, subsequently cotreated with LPS for 22 h, WB was used to measure iNOS and COX2 protein expression. (F) The relative expression levels of iNOS and COX2. (G, H) The mRNA expression of iNOS, COX2 in THP1 cells. (I-K) The mRNA expression of IL-1β, IL-6, and TNF-α in RAW264.7 cells. (L-N) The mRNA levels of IL-1β, IL-6, and TNF-α in THP1 cells. Data were displayed as mean ± SD (n=3). $^{\#}p < 0.05$, $^{\#\#}p < 0.01$ versus the control group. $^{*}p < 0.05$, $^{**}p < 0.01$ versus the LPS-treated group.

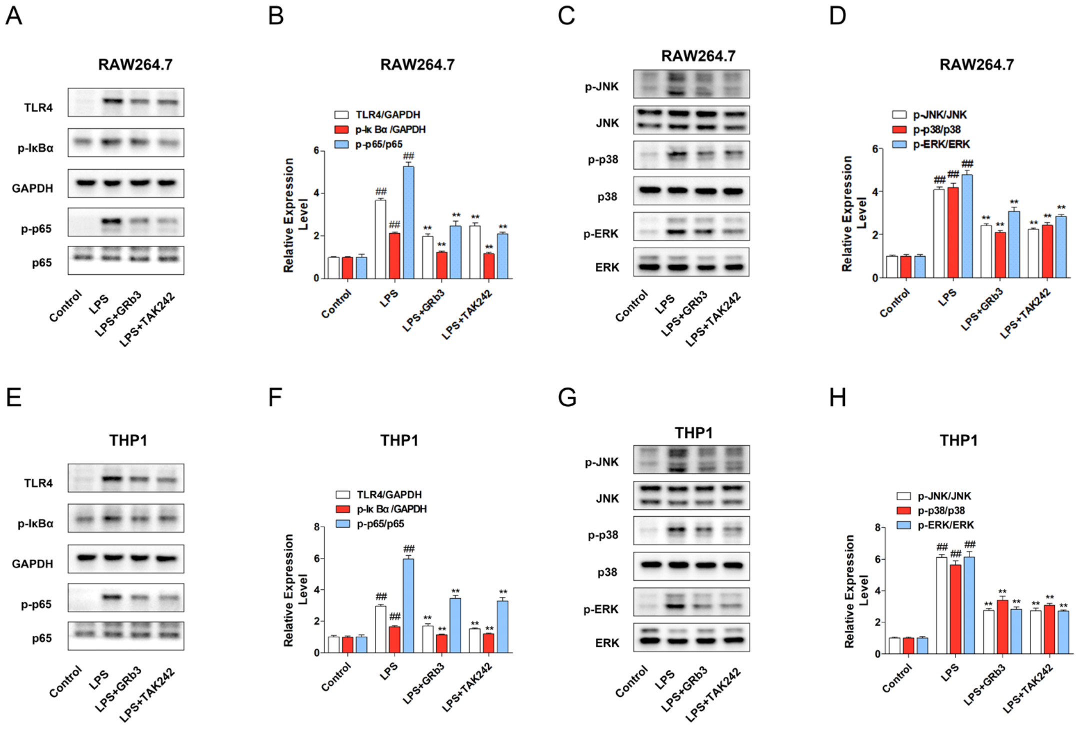


**Supplementary Figure S2. The effects of GRb3 and TAK242 on TLR4/NF-κB/MAPK signaling in RAW264.7 cells and THP1 cells.** (A) After incubation with 100 μM GRb3 or 100 nM TAK242 for 24 h, RAW264.7 cells were cotreated with LPS for 1 h, followed by WB to test TLR4, p-IκBα, and p-p65 expression. (B) The relative expression levels of TLR4, p-IκBα, and p-p65. (C) Effects of GRb3 and TAK242 on expression of p-JNK, p-p38, and p-ERK in RAW264.7 cells, and their relative expression levels were standardized to JNK, p38, and ERK (D). (E) THP1 cells were incubated with 100 μM GRb3 or 100 nM TAK242 for 24 h, subsequently cotreated with LPS for 1 h, WB was used to measure TLR4, p-IκBα, and p-p65 protein expression. (F) The relative expression levels of TLR4, p-IκBα, and p-p65 in THP1 cells. (G) Effects of GRb3 and TAK242 on expression

of p-JNK, p-p38, and p-ERK in THP1 cells, and their relative expression levels were standardized to JNK, p38, and ERK (H). Data were dispalyed as mean ± SD (n=3). $^{\#}p < 0.05$, $^{\#\#}p < 0.01$ versus the control group. $^{*}p < 0.05$, $^{**}p < 0.01$ versus the LPS-treated group.

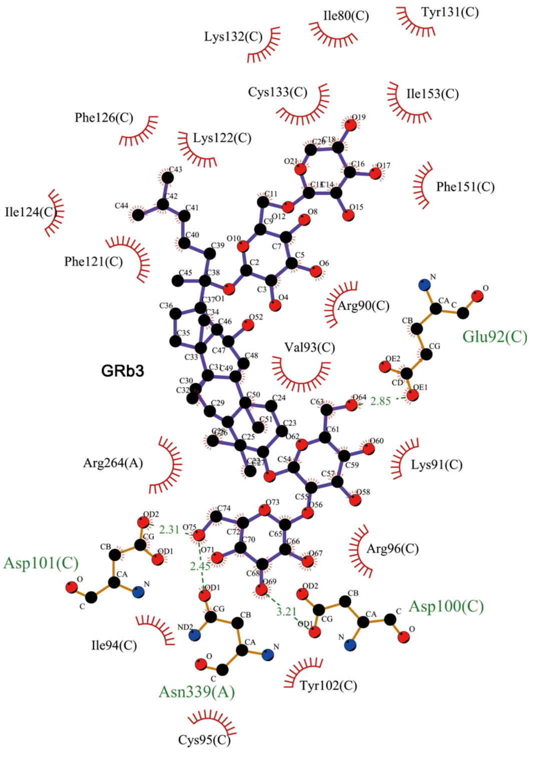


MD2-GRb3

**Supplementary Figure S3. The docking amino acid residues of MD2 to GRb3.**
